# Data on prognostication models comparison for neurological recovery after cardiac arrest using proton chemical shift imaging (^1^H-CSI)

**DOI:** 10.1016/j.dib.2018.10.066

**Published:** 2018-10-25

**Authors:** Hervé Quintard, Lionel Velly, Salah Boussen, Xavier Chiosi, Marie-Eve Amoretti, Elodie Cervantes, Carole Ichai

**Affiliations:** a Université Côte d׳Azur, CHU de Nice, Department of Anaesthesiology and Critical Care Medicine, Hôpital Pasteur 2, Nice, France; b CNRS, UMR 7275, Sophia Antipolis, France; c Aix Marseille University, Department of Anaesthesiology and Critical Care Medicine, University Hospital La Timone, Marseille, France; d Aix Marseille University, Institut de Neuroscience de la Timone (INT), Marseille, France; e Aix Marseille University, Laboratoire de Biomécanique Appliquée (IFSTTAR), Marseille, France; f Université Côte d׳Azur, CHU de Nice, Radiology Department, Hôpital Pasteur 2, Nice, France

## Abstract

We report in this data article the statistical comparison of three models for neurological prognostication 6 months after cardiac arrest: M1 associated SAPS II and coma Glasgow score at MRI, M2 associated SAPS II, coma Glasgow score, and FLAIR-DWI “deep gray nuclei”score, M3 associated SAPS II, coma Glasgow score, FLAIR-DWI “deep gray nuclei”score, and Lenticular cores NAA/Cr ratio. These data are related to “Value of assessment of multivoxel proton chemical shift imaging to predict long term outcome in patients after out-of-hospital cardiac arrest: A preliminary prospective observational study” (Quintard et al., 2018) [1].

**Specifications table**

**Table:** 

Subject area	*Medicine*
More specific subject area	*Neurological prognosis after cardiac arrest*
Type of data	*Graph and tables*
How data was acquired	*Multivoxel 1H-CSI values*
	*Structural magnetic resonance imaging (MRI) sequences (fluid-attenuated inversion recovery and diffusion-weighted imaging).*
Data format	*Statistical*
Experimental factors	*The MRI data were collected 7 days after cardiac arrest*
Experimental features	*Three incremental models were compared according neurological prognosis 6 months after cardiac arrest*
Data source location	*University intensive care unit Nice, France*
Data accessibility	*Data is with this article*
Related research article	*Value of Assessment of Multivoxel Proton Chemical Shift Imaging to Predict Long Term Outcome in Patients after Cardiac Arrest: A Prospective Observational Study. Quintard H, Velly L, Boussen S, Chiosi X, Amoretti ME, CervantesE, Ichai C. Resuscitation. 2018 Sep 21. pii: S0300–9572(18)30885-2. doi:*
	10.1016/j.resuscitation.2018.09.007*. r*

**Value of the data**

• The data presented here was used to develop an implemental model prognosis after cardiac arrest in adult.
• The significance of the data is potentially helpful for predicting recovery after cardiac arrest.

## 1 Data

**Fig. 1:**
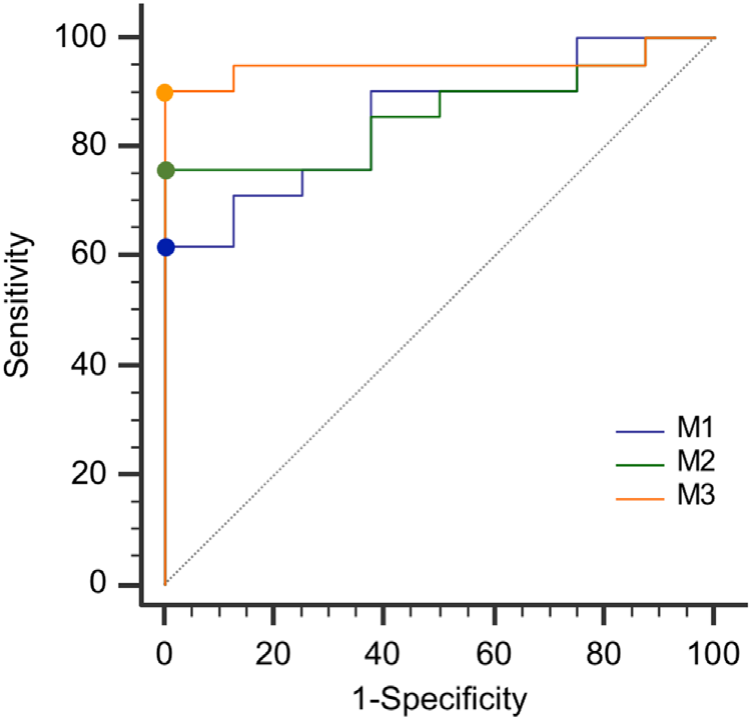
Receiver-Operating-Characteristic curves for the three multivariate logistic regression models. In the first model (M1), were considered: SAPS II and Glasgow Coma Scale at MRI. In the second model (M2), qualitative MRI variable (FLAIR-DWI “deep gray nuclei” score) was added to those in the first model. In the third model (M3), predictive quantitative MRI variable (Lenticular cores NAA/Cr ratio) was added to the second model.

Twenty-nine patients were included in the analysis [1]. The neurologic follow-up, evaluated as Cerebral Performance category (CPC, Additional File 1) was specified in the protocol to be at 180 ± 14 days, but the time to follow-up was in some cases several weeks longer for logistic reasons. Twenty-one patients had a bad outcome (CPC 3–5) at 6 months and 9 a favorable one (CPC 1–2) (Table 1). Data report multivariate analysis (Table 2) of 3 prognosis models: M1 associated SAPS II and Glasgow coma score at MRI, M2 associated SAPS II, coma Glasgow score, and FLAIR-DWI “deep gray nuclei” score, M3 associated SAPS II, coma Glasgow score, FLAIR-DWI “deep gray nuclei” score, and Lenticular cores NAA/Cr ratio. Receiver-Operating-Characteristic curves for the three multivariate logistic regression models were realized (Fig. 1).

**Table 1:** Outcomes in the cohort.

			**All patients**	Favorable outcome at follow-up* (CPC 1–2)	Unfavorable outcome at follow-up* (CPC 3–5)
			(*N* = 29)	(*N* = 8)	(*N* = 21)
**Variables**					
**Best numerical, Cerebral Performance Categories (CPC) during trial**					
Category — no. (%)					
1			6 (21)	5 (62)	1 (5)
2			6 (21)	3 (38)	3 (14)
3			0 (0)	0 (0)	0 (0)
4			17 (58)	0 (0)	17 (81)
5			NA	NA	NA
**CPC at ICU discharge**					
Category — no. (%)					
1			1 (3)	0 (0)	1 (5)
2			10 (35)	7 (88)	3 (14)
3			1 (3)	1 (12)	0 (0)
4			0 (0)	0 (0)	0 (0)
5			17 (59)	0 (0)	17 (81)
CPC at follow-up*					
Category — no. (%)					
1			5 (17)	5 (62)	0 (0)
2			3 (10)	3 (38)	0 (0)
3			0 (0)	0 (0)	0 (0)
4			1 (3)	0 (0)	1 (5)
5			20 (69)	0 (0)	20 (95)
**Time of survival if dead**					
Median			16	–	16
Interquartile range			12–40	–	12–40

The neurologic follow-up was specified in the protocol to be at 180 ± 14 days, but the time to follow-up was in some cases several weeks longer for logistic reasons. † Cause of death missing in four cases in the derivation cohort and in one case in the validation cohort. ICU denotes intensive care unit.

**Table 2:** Multivariate analysis in the cohort.

		***Multivariate logistic regression***			
	Unit (UI)	Estimated Coefficient	Standard Error	Odds Ratio *(95% Confidence Interval)*	*P* Value
**SAPS II - Glasgow Coma Scale at MRI**					
SAPS II	Per UI increase	0.04	0.03	1.04 (1.00 – 1.10)	0.17
Glasgow Coma Scale at MRI	Per UI increase	-0.2	0.1	1.25 (1.01–1.56)	0.04
**SAPS II - Glasgow Coma Scale at MRI - FLAIR-DWI “deep gray nuclei” score**					
SAPS II	Per UI increase	0.04	0.03	1.04 (0.98 – 1.10)	0.16
Glasgow Coma Scale at MRI	Per UI increase	-0.15	0.1	0.85 (0.68 – 1.10)	0.20
FLAIR-DWI “deep gray nuclei” score	Per UI increase	0.05	0.04	1.05 (0.96 – 1.15)	0.25
**SAPS II - Glasgow Coma Scale at MRI - FLAIR-DWI “deep gray nuclei” score- NAA/Cr Lenticular cores**					
SAPS II	Per UI increase	0.05	0.03	1.05 (0.98 – 1.12)	0.14
Glasgow Coma Scale at MRI	Per UI increase	-0.05	0.2	0.95 (0.70 – 1.30)	0.76
FLAIR-DWI “deep gray nuclei” score	Per UI increase	0.08	0.06	1.09 (0.96 – 1.23)	0.17
NAA/Cr Lenticular cores	Per UI decrease	-4.4	2.5	0.01 (0.00 – 1.70)	0.08

DWI, diffusion-weighted imaging; FLAIR, fluid-attenuated inversion recovery; MRI, Magnetic Resonance Imaging; NAA/Cr, N-acetyl aspartate over creatine ratios and SAPS II, Simplified Acute Physiology Score II.

## 2 Experimental design, materials, and methods

MR imaging was performed mean 7 days after CA, according to tolerance of transport of such patients. Structural MRI and 1H-MRS, were performed on 1.5-T Signa HDxt imager (GE Healthcare, Milwaukee, WI, USA). Pulse sequences included a T2-weighted sequence (axial; slice thickness, 5 mm; TR/TE, 6400/105 ms; bandwidth, 122 Hz; flip angle, 90°; matrix, 512 × 320; field of view (FOV), 240 mm), T2*-weighted sequence (axial; slice thickness, 5 mm; TR/TE, 640/18 ms; bandwidth, 57 Hz; flip angle, 20°; matrix, 352 × 224; FOV, 250 mm), fluid-attenuated inversion recovery (FLAIR; axial; slice thickness, 5 mm; TR/TE/TI, 9000/160/2250 ms; bandwidth, 122 Hz; flipangle, 90°; matrix, 256 × 192; FOV, 250 mm), and diffusion-weighted imaging (DWI; axial; slice thickness, 3 mm; TR/TE, 6150/102 ms; max b value of 1000 s/mm^2^; bandwidth, 1953 Hz; flip angle, 90°; matrix, 128 × 128; FOV, 250 mm). Structural MRI acquisition took about 20 min, depending on the number of slices required to cover the brain. Multivoxel 1H-CSI was acquired using the point-resolved proton spectroscopy sequence (PRESS) with the following parameters: slice thickness, 20 mm; TR/TE, 1000/144 ms; matrix, 16 × 16; FOV, 240 mm; number of excitations, 1. The axial 1H-MRS covering a region from the head of striatum to the thalamus. This acquisition took 11 min. The MR images were retrieved in DICOM (Digital Imaging and Communications in Medicine) format from picture archiving and communication system servers at the hospitals (Table 3).

**Table 3:** Prognostic values of significant variables of the patients without a limitation or withdrawal (n=14) of care decision in the cohort.

**Variables**	**ROC**_**AUC**_***(95% confidence interval)***	**Optimal Cutoff**	**Specificity**		**Sensitivity**	**Predictive Positive Value**			**Negative Predictive Value**
			*Expressed in percent (95% confidence interval)*						
*Clinical and biological criteria*									
Serum lactate at H24	0.63 (0.35-0,87)	> 7.25	100 (63–100)	0 (0–46)			–	57 (29–82)	
Glasgow Coma Scale at MRI	0.56 (0.28–0.82)	≥ 15	100 (63–100)	0 (0–46)			–	57 (29–82)	
*Qualitative Magnetic Resonance Imaging (MRI) variables*									
FLAIR-DWI “deep gray nuclei” score	0.65 (0.35–0.87)	> 8	100 (63–100)	67 (34–78)			100 (16–100)	67 (35–90)	
*Quantitative Magnetic Resonance Imaging (MRI) variables*									
NAA/Cr Lenticular cores	0.85 (0.57–0.98)	≤ 1.30	100 (63–100)	83 (36–100)			100 (48–100)	89 (52–100)	

DWI, diffusion-weighted imaging; FLAIR, fluid-attenuated inversion recovery; NAA/Cr, N-acetyl aspartate over creatine ratios and SAPS II, Simplified Acute Physiology Score II.

† Sensitivity significantly different than the one of the NAA/Cr Lenticular cores (*P*< 0.05).

### 2.1 Image processing

Brain MR images were reviewed by a board-certified blinded radiologist (ME.A, with 10 years of postfellowship experience) to evaluate for structural abnormalities. Participants with any evidence of territorial stroke of any age, intracranial hemorrhage, or intracranial mass lesions were excluded; in addition, any subject with head motion greater than 2 mm across the imaging session was excluded. MR imaging morphologic analysis was accomplished using a validated score on the basis of a visual rating. In the FLAIR-DWI scoring (Additional file 2) proposed by Hirsch et al. [2] developed in a similar population with CA, the anatomic location and degree of signal abnormality on FLAIR images and DWI sequences is rated from 0 (normal) to 4 (severe), and composite tissue signal change is then calculated in cortical, basal ganglia and thalamus, brainstem, and cerebellar structures. The sum of scores in cortical, basal ganglia, and all structures is referred to as the “cortex”, “deep gray nuclei”, and “overall score”, respectively. The sum or all scores recorded by using FLAIR and DWI is known as the DWI-FLAIR score, respectively [2].

Multivoxel 1H-CSI data processing was performed by an expert radiologist allowing to measure of the area under the curve by standard manufacturer software dedicated to MR spectroscopy post-processing (Advantage Windows for General Electric). The volume of interest was placed carefully to avoid contact with cerebral spinal fluid on non-angled FLAIR images after coregistration of spectroscopic data and FLAIR volume. The spectra were analyzed for determining the concentration of metabolites in the lenticular cores (right and left side) and thalami (right and left side): NAA (at 2.0 ppm), Cho (at 3.2 ppm) and Cr (at 3.0 ppm). The quality of the selected spectra was visually inspected and was considered acceptable only if Cho and Cr signals were clearly separated. Spectra was also rejected if MR spectroscopists detected artefacts such as large baseline distortions, exceptionally broadened metabolite peaks, insufficient removal of the water line, large phase errors, and signals originating from outside the voxel as conventionally used in clinical practice. In all cases, peak surface area was used to calculate metabolite ratios for metabolite quantification. The worst value of bilateral lenticular cores and thalami were analyzed except in cases of some non-interpretable voxels. In this case, the sole side with spectra of good quality was considered.

### 2.2 Calculation of odds ratios

Because there were categorical and continuous variables with different units, odds ratios (with 95%CI) were computed by taking the exponent of the absolute value of the estimated parameters (and 95% CI), the latter being multiplied by a factor that accounts for the unit used (i.e., 0.1 in the case of 1H-MRS parameters). We tested the null hypothesis of an estimated parameter being equal to zero with the use of the likelihood ratio test (LR test) with one degree of freedom. This corresponds to the null hypothesis of an odds ratio being equal to 1, i.e., no predictive value. Hence, a variable was considered to be predictive if *P*< 0.05.
