# Supplementary material for: Data on prognostication models comparison for neurological recovery after cardiac arrest using proton chemical shift imaging (1H-CSI)
Source: Data Brief. 2018 Oct 25;21:893–8. doi: 10.1016/j.dib.2018.10.066 (PMC6222258; doi:10.1016/j.dib.2018.10.066)
Supplement: Supplementary file 2 — Supplementary material [file mmc2.docx]

| 1. **Score** |  | **Description** | |  |
| --- | --- | --- | --- | --- |
|  |  |  | |  |
| 1 | (Good Cerebral Performance) | | Conscious. Alert, able to work and lead a normal life. May have minor psychological or neurological deficits (mild dysphasia, non-incapacitating hemiparesis, or minor cranial nerve abnormalities). | |
| 1. 2 | (Moderate Cerebral Disability) | | Conscious. Sufficient cerebral function for part-time work in sheltered environment or independent activities of daily life (dressing, traveling by public transportation, and preparing food). May have hemiplegia, seizures, ataxia, dysarthria, dysphasia, or permanent memory or mental changes. | |
| 1. 3 | (Severe Cerebral Disability) | | Conscious. Dependent on others for daily support because of impaired brain function (in an institution or at home with exceptional family effort). At least limited cognition. Includes a wide range of cerebral abnormalities from ambulatory with severe memory disturbance or dementia precluding independent existence to paralytic and able to communicate only with eyes, as in the locked-in syndrome. | |
| 1. 4 | (Coma, Vegetative State) | | Not conscious. Unaware of surroundings, no cognition. No verbal or psychological interactions with environment. | |
| 1. 5 | (Death) | | Certified brain dead or dead by traditional criteria. | |

# Additional file 1: Glasgow-Pittsburgh Cerebral Performance Categories (CPC){Citation}
